# Arthroscopic removal of a solitary osteochondroma interfering with the podotrochlear apparatus in a foal

**DOI:** 10.1111/vsu.13522

**Published:** 2020-10-14

**Authors:** Machiel P. Ysebaert, Jessica P. Johnson, Ghazanfar Abbas, Paulo Henrique Cavalcante, Rodney King, Masa Oikawa, Sarah Puchalski, Florent David

**Affiliations:** ^1^ Equine Veterinary Medical Center A Member of Qatar Foundation Al Shaqab street, Al Rayyan Doha Qatar; ^2^ College of Health & Life Science Hamad Bin Khalifa University, A Member of Qatar Foundation Education City Doha Qatar; ^3^ Al Shaqab Breeding & Show A Member of Qatar Foundation Al Shaqab street, Al Rayyan Doha Qatar; ^4^ Puchalski Equine Diagnostic Imaging Petaluma California USA

## Abstract

**Objective:**

To report the diagnostics, surgical treatment, and outcome of a juvenile foal with solitary osteochondroma (SO) interfering with the podotrochlear apparatus.

**Study design:**

Case report

**Animal:**

One 36‐day‐old Arabian colt.

**Methods:**

Clinical, radiographic, ultrasonographic, computed tomographic, and histopathologic examinations were required to characterize and treat an SO located at the palmar aspect of the diaphysis of the second phalanx of the left forelimb. This SO caused severe distal interphalangeal joint (DIPJ) inflammation, marked interference with the podotrochlear apparatus, and associated lameness.

**Results:**

Despite the small size of the foal's foot, complete resection of the SO was possible via palmar DIPJ arthroscopy by using motorized equipment. Full resolution of the lameness was achieved within 3 months of surgery.

**Conclusion:**

Atypical SO located on the palmar aspect of the second phalanx can cause marked nonseptic inflammation of the DIPJ and interference with the podotrochlear apparatus and should be considered among the differential diagnoses for severe lameness in juvenile foals. Arthroscopic resection of the SO led to an excellent outcome.

## 1 INTRODUCTION

Solitary osteochondromas (SO) are benign bone masses that can occur in any bone that undergoes endochondral ossification. In horses, they are most commonly found in the distal radius^1^, ^2^ and are defined as cartilage‐capped osseous projections on the external surface of the bone.^3^ They are thought to originate from an accidental separation and migration toward the diaphysis of a portion of physeal cartilage during skeletal development. This aberrant subperiosteal physeal cartilage establishes a separate focus of endochondral ossification, and this results in an outgrowth from the metaphysis, or extremity of the diaphysis, that ceases growing at the time of skeletal maturity.^4^ Although they develop in growing horses, SO in the distal radius are typically detected in equine adults 3 to 12 years old due to the expansive nature of the osseous herniation impinging on the adjacent soft tissue structures.^5^ Other less frequently reported sites for SO are distal caudal tibia, cranial aspect of distal radius, rib, vertebra, scapula, dorsal aspect of the proximal phalanx, and flat bones.^4^ Solitary osteochondromas causing clinical signs in foals are rare, and, to the best of our knowledge, only two cases have been described.^6^, ^7^ In one previous case report, a 2‐month‐old foal with an SO located on the second phalanx (P2) was described, but this foal was euthanized because of poor prognosis.^7^ In Arabian horses, the P2 physis is open at birth, and complete closure occurs at approximately 7 months of age. For the distal radial physis, full closure is observed at approximately 24 months.^8^ These differences in physeal closure times likely explain why SO in different locations can become symptomatic at different ages.

Severe lameness in young foals is usually attributed to an infectious systemic cause with local manifestations such as septic arthritis/physitis or noninfectious causes such as congenital malformations, fractures, or severe forms of developmental orthopedic disease.In this report, we describe a juvenile Arabian colt with severe forelimb lameness in which P2 SO interfering with the podotrochlear apparatus was diagnosed by using radiography, ultrasonography, computed tomography (CT)‐arthrography, and histopathology and was treated successfully by using a minimally invasive surgical technique.

## 2 MATERIALS AND METHODS

### 2.1 History

All work was performed at the Equine Veterinary Medical Center, Doha, Qatar.

A 36‐day‐old Arabian colt was referred to the Equine Veterinary Medical Center with a grade 4 of 5 (AAEP scale^9^) left thoracic limb (LF) lameness. The lameness was temporarily abolished with an abaxial sesamoidean nerve block performed at the level of the base of the proximal sesamoid bones by the referring veterinarian. Radiographic examination revealed exostosis at the mid‐distal palmar aspect of P2, immediately proximal to the distal sesamoid bone (DSB). The foal had been given firocoxib (0.1 mg/kg orally once daily) for 1 week, but the lameness persisted.

### 2.2 Clinical findings

The colt had moderate effusion of the LF distal interphalangeal joint (DIPJ). Digital pulses were moderately increased, and pain was evident when the LF pastern region was palpated and passive flexion of the digit was performed. Hoof testing of this small foot (88 × 43 × 69 mm) was negative.

On dynamic examination, a grade 4 of 5 LF lameness was observed. The colt exhibited a preference for canter and appeared to avoid trotting. Because of the foal's young age, flexion tests were not performed.

Bloodwork revealed a mild leucopenia, mild hypoproteinemia, and a mild hypoalbuminemia.

### 2.3 Radiography

**FIGURE 1:**
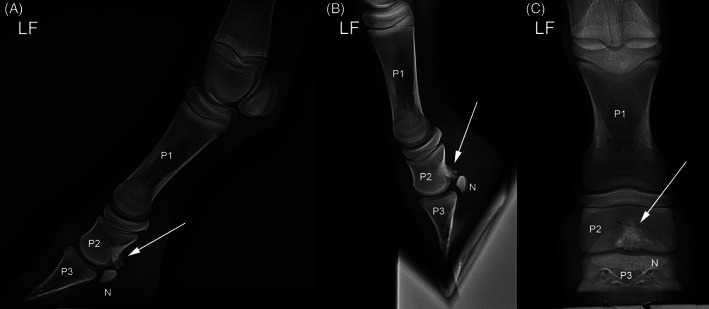
Weight‐bearing (A) and flexed (B) lateromedial and weight‐bearing dorsopalmar (C) radiographic views of the LF digit at time of diagnosis. The arrow indicates a spiculated osseous mass originating from the palmar cortex of P2, distant from the (proximal) growth plate and interfering with the articular surface of the distal sesamoid bone in both weight bearing and flexed positions. LF, left thoracic limb; N, distal sesamoid bone; P1, first phalanx; P2, second phalanx; P3, third phalanx

A sagittal, spiculated osseous mass at the palmar and mid‐distal aspect of LF P2, immediately proximal to the DSB, was evident on the lateromedial and dorsopalmar views (Figure 1A‐C). There was markedly increased bone opacity dorsal to this osseous mass. Screening radiographs of the other limbs did not reveal any additional exostoses.

### 2.4 Ultrasonography

**FIGURE 2:**
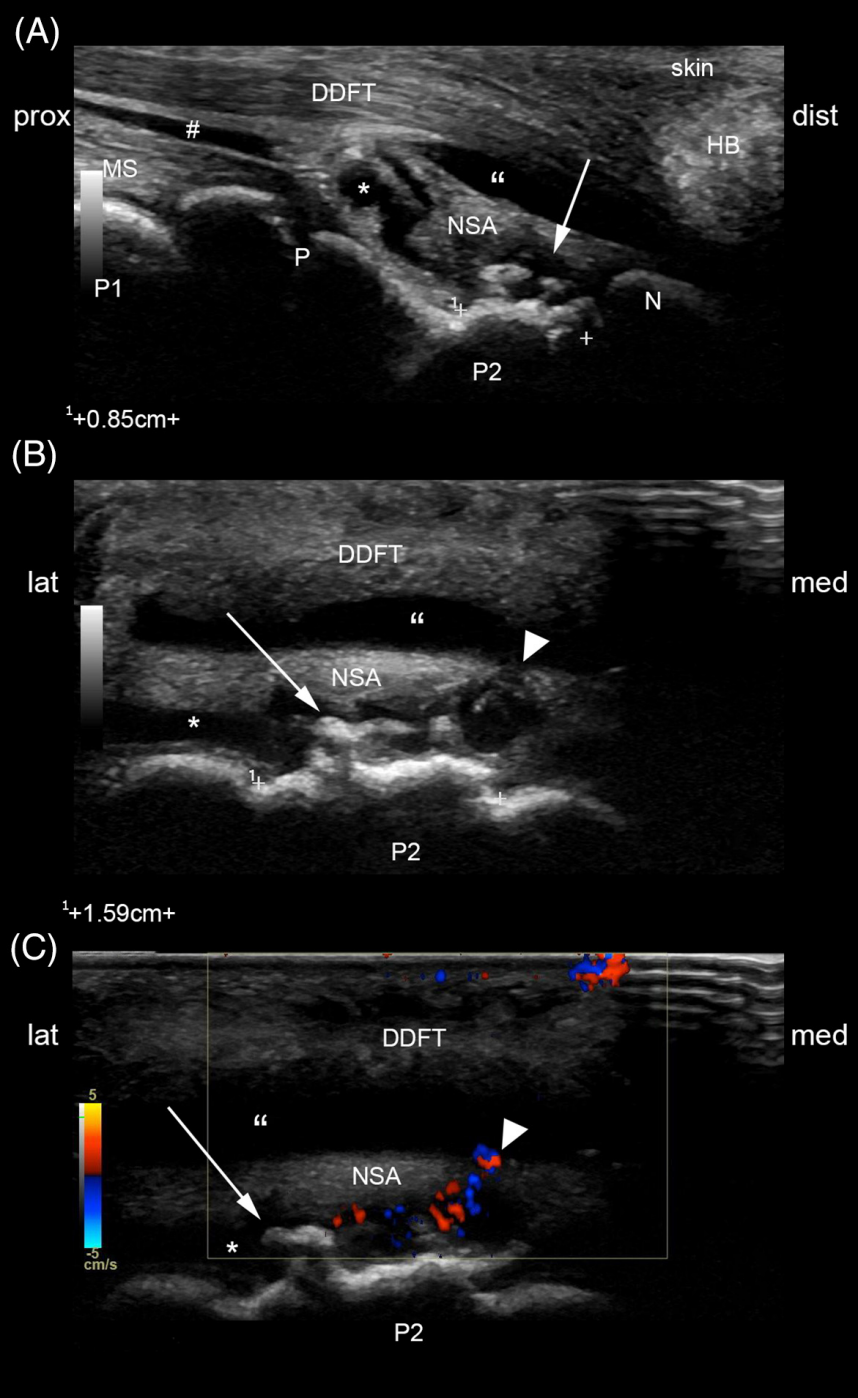
Palmar longitudinal (A) and transverse (B) B‐mode and transverse color Doppler (C) ultrasonographic images obtained with a 12‐MHz linear transducer. Spiculated osseous mass (arrow) originating from the palmar aspect of P2 impinges on the NSA. A communication (arrowheads) between the distal interphalangeal joint and the navicular bursa, the latter being markedly effused, is suspected on the transverse B‐Mode and confirmed on color Doppler ultrasonographic image. *, distal interphalangeal joint proximal palmar recess; DDFT, deep digital flexor tendon; dist, distal; HB, heel bulb; lat, lateral; med, medial; MS, middle scutum; N, distal sesamoid bone; NSA, navicular suspensory apparatus; P, P2 physis; P1, first phalanx; P2, second phalanx; #, digital flexor tendon sheath; prox, proximal; ", navicular bursa proximal recess

The foal was restrained, and an ultrasound examination was performed (GE Logiq E9 XDclear, 12 MHz linear and 6 MHz curvilinear transducers; GE Healthcare, Piscataway, New Jersey). A large volume of hypoechogenic fluid, indicating effusion and synovitis, was appreciated in the dorsal recess of the LF DIPJ. A large (15.9 × 8.5 mm) spiculated osseous mass was noted to protrude from the palmar aspect of P2, surrounded by hypoechogenic fluid (Figure 2A,B). The fluid collection was well delineated, extending proximally up to the level of P2 physis. Synovial proliferation was noted, with thickening of the DIPJ joint capsule. The proximal recess of the navicular bursa (NB) was also distended with hypoechogenic fluid, highlighting the suspensory ligament of the DSB. This ligament appeared perforated on its medial aspect, and a small communication between the palmar and proximal recess of DIPJ and the proximal recess of the NB was confirmed on Doppler ultrasonographic image (Figure 2C). The spikes of the osseous mass did not appear to contact the deep digital flexor tendon (DDFT) in non–weight‐bearing position.

### 2.5 Computed tomography

**FIGURE 3:**
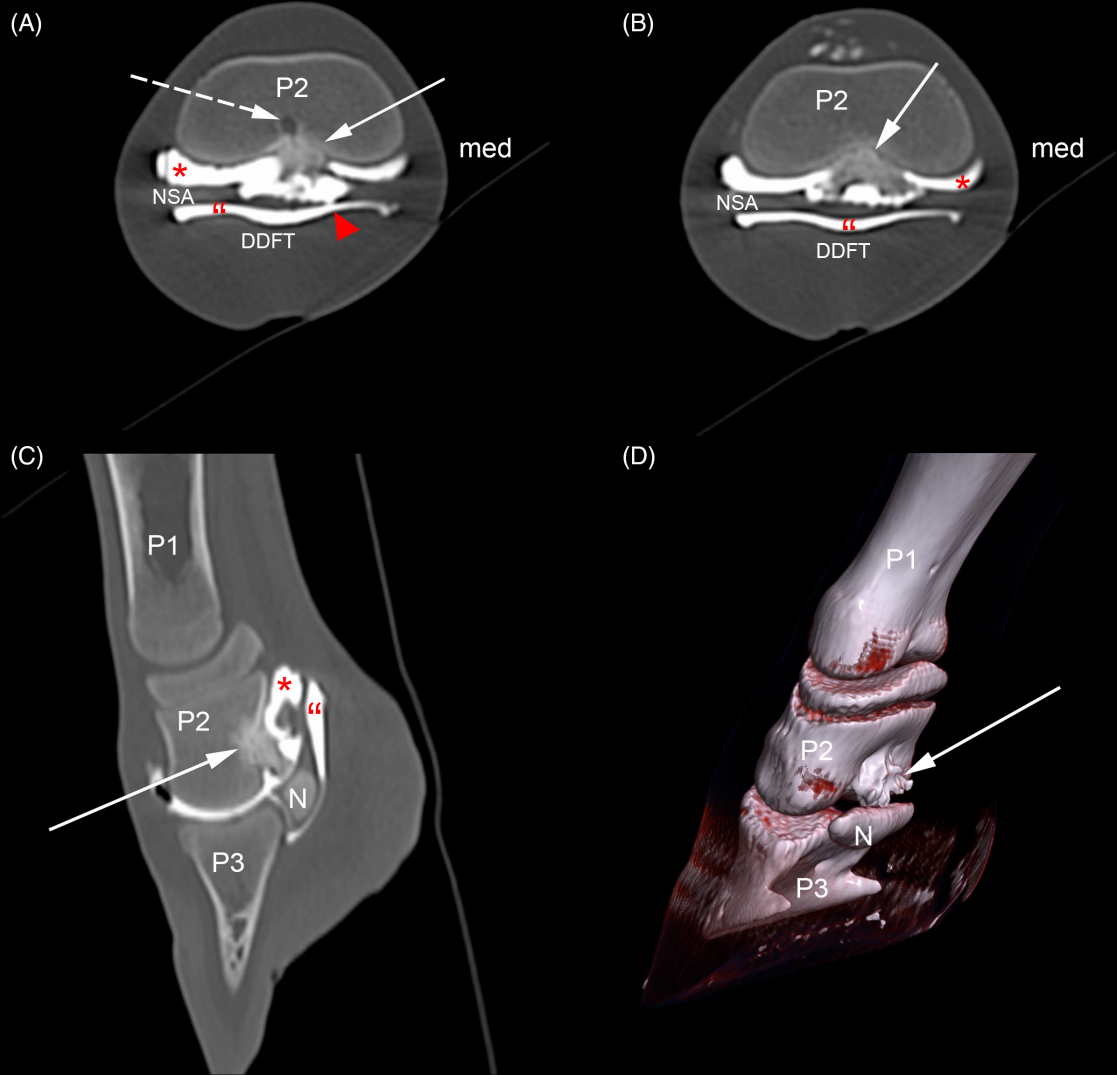
Computed tomography‐arthrogram images. Transverse plane, at the level of mid‐ (A) and distal (B) P2; sagittal plane (C); three dimensional reconstruction (D) SO (arrow) originates from the palmar cortex of P2, composed of dense spiculated bone, surrounded by synovial fluid (contrast agent) confirming its articular location. SO impinges the NSA. A nutrient foramen (dashed arrow) can be seen within the dorsal aspect of the SO, providing evidence of its extension into the medullary cavity. Arrowhead, communication between distal interphalangeal joint and navicular bursa; *, distal interphalangeal joint proximal palmar recess; DDFT, deep digital flexor tendon; med, medial; N, distal sesamoid bone; NSA, navicular bone suspensory apparatus; P1, first phalanx; P2, second phalanx; P3, third phalanx; ", navicular bursa proximal recess; SO, solitary osteochondroma

The foal was anesthetized and placed in sternal recumbency for CT examination of both front digits (Somatom Definition AS, 128‐slice; Siemens, Munich, Germany). One‐millimeter helical images processed by using a high‐frequency convolution kernel were acquired from the carpus distally. Computed tomographic images revealed an irregular, heterogeneous mineral mass (~1200 Hounsfield units [HU]) hyperdense to cortical bone (1100 HU) extending in both palmar and dorsal directions from mid‐diaphyseal cortical bone of P2. The palmar component displaced the suspensory ligament of the DSB in a palmar direction. The dorsal component was closely associated with the nutrient foramen in the medullary cavity of P2 (Figure 3A‐D).

Distal interphalangeal joint CT‐arthrography (dorsal approach, 3 mL iohexol at 300 mg I/mL) revealed that the osseous mass was covered by a thin layer of soft tissue surrounded by the contrast laden synovial fluid (Figure 3A‐C 3B), providing evidence of an intrasynovial location in the proximal palmar recess of the DIPJ and confirming DIPJ/NB communication.

### 2.6 Cytology

The synovial fluid aspirated at the time of CT‐arthrography was hemorrhagic, with total protein (28 g/L), white blood cell count (0.928 × 10^9^/L), and neutrophils (50%), was consistent with a nonseptic arthritis of the DIPJ, so culture was not deemed required. The foal received meloxicam (0.6 mg/kg orally once daily) to maintain comfort levels until surgery 48 hours later.

### 2.7 Surgical procedure

The foal received ceftiofur 2.2 mg/kg IV and flunixin meglumine 1.1 mg/kg IV prior to induction. Anesthesia induction was achieved with diazepam 0.06 mg/kg IV and ketamine 2.2 mg/kg IV, and anesthesia was maintained by using controlled ventilation with isoflurane. Aseptic preparation of the LF foot was performed^10^ in dorsal recumbency, and the solar surface was fixed to a metallic bar with adhesive tape. The peripodal skin was then prepared aseptically, and iodine tincture was applied to the horn. An abaxial sesamoidean nerve block was performed with bupivacaine for perioperative analgesia.

The DIPJ was distended maximally with 20 mL of sterile saline with a 20‐gauge needle inserted dorsally and 5 mm proximal to the coronary band. The first arthroscopic portal was made at the most proximal, palmar, and lateral aspect of the DIPJ, abaxial to the lateral digital neurovascular bundle, approximately 1.5 cm proximal to the ipsilateral ungular cartilage. A 5.8‐mm outer diameter (OD) self‐locking arthroscopic cannula and a conical obturator were then carefully inserted into the joint in a medial‐45° distal‐20° dorsal direction.^11^ A 4‐mm diameter 30° fore‐oblique 18‐cm long arthroscope (Hopkins II; Karl Storz, Tuttlingen, Germany) was introduced, and correct placement within the synovial space was verified.

**FIGURE 4:**
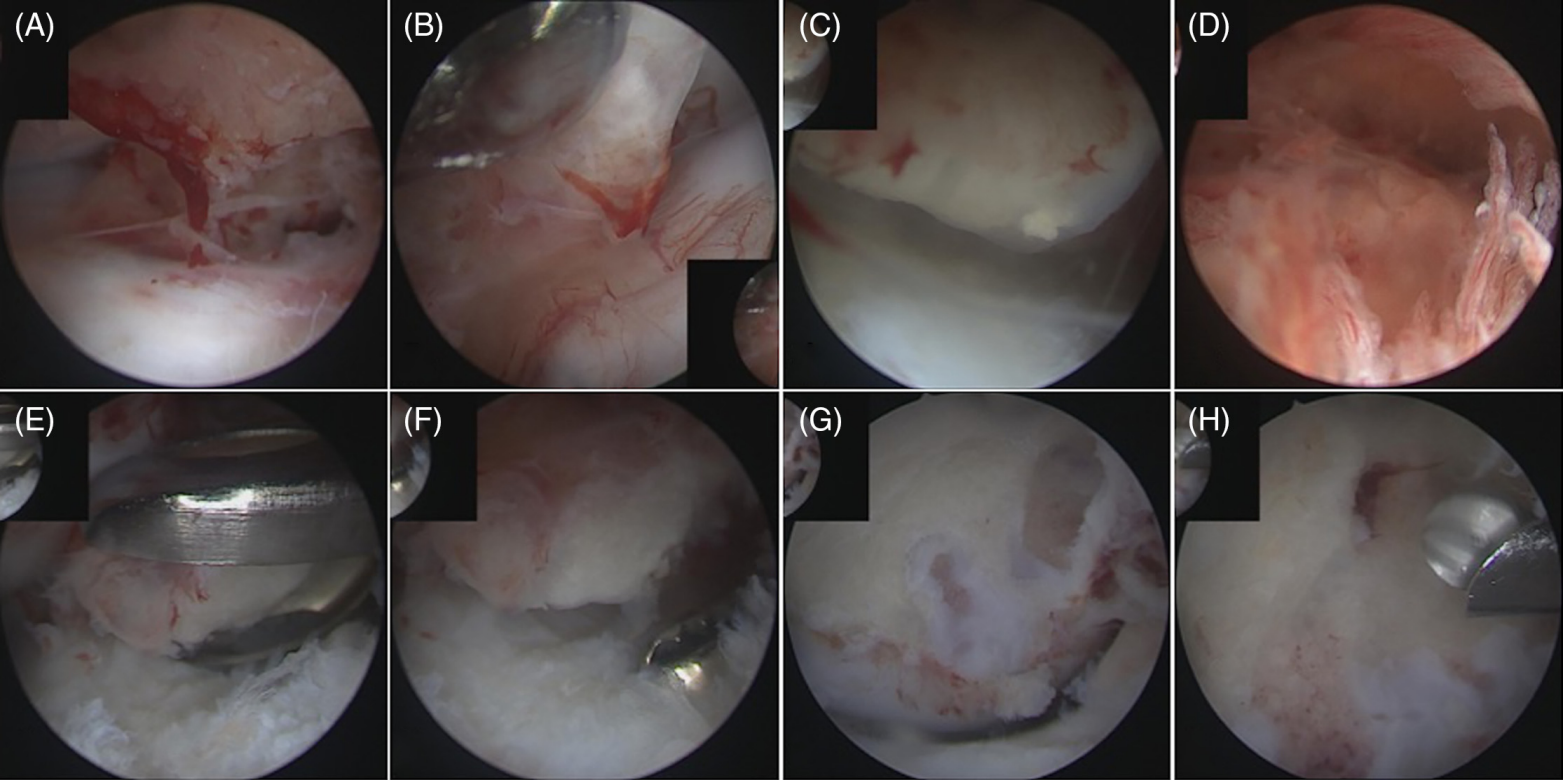
Arthroscopic images (also see Supporting Information videos) taken during exploration (A‐D) of the palmar (proximal) recess of the distal interphalangeal joint and removal of solitary osteochondromas (E‐H). The spiculated osseous mass (A,B), covered with fibrin and recent blood clots, protrudes toward the joint capsule and the navicular bone suspensory apparatus. A marked synovitis is present (D). When it is closely examined (C), it can be seen that the mass is covered by cartilage with ossification visible within this layer. Bone biopsies (E) and debridement of the proliferated synovium (F) were performed prior to debridement of the osseous mass with a bone burr (G,H). The nutrient foramen became visible close to the end of debridement (H)

The joint fluid was hemorrhagic at initial exploration (Figure 4A‐D; also see Supporting Information videos). The irregular osseous mass was identified at the palmar aspect of P2 and appeared to be covered by cartilage and fibrin. The mass was noted to be protruding into the suspensory apparatus of the DSB. The proximal edge of the DSB was visible only along its lateral margin.

On the medial aspect of the joint, an instrumental portal was created as proximally as possible, abaxial to the medial neurovascular bundle. A synovial resector (reusable 3.5‐mm aggressive cutter; Karl Storz) was used to remove the proliferated synovium as well as the fibrin and blood clots overlying the bone mass. This improved visibility of the margins of the SO, and a full thickness tear through the suspensory apparatus of the DSB could be appreciated, revealing a small communication with the NB medially. The DDFT was visible through the tear.

Initial parts of the osseous protuberance were removed by using rongeurs and submitted for histopathology (Figure 4E). Thereafter, the bulk of the mass was progressively resected by using a motorized bone burr (reusable 4.2‐mm round burr; Karl Storz) until healthy underlying bone was encountered (Figure 4F‐H). High‐volume lavage was performed simultaneously to remove the resultant chondro‐osseous debris.

**FIGURE 5:**
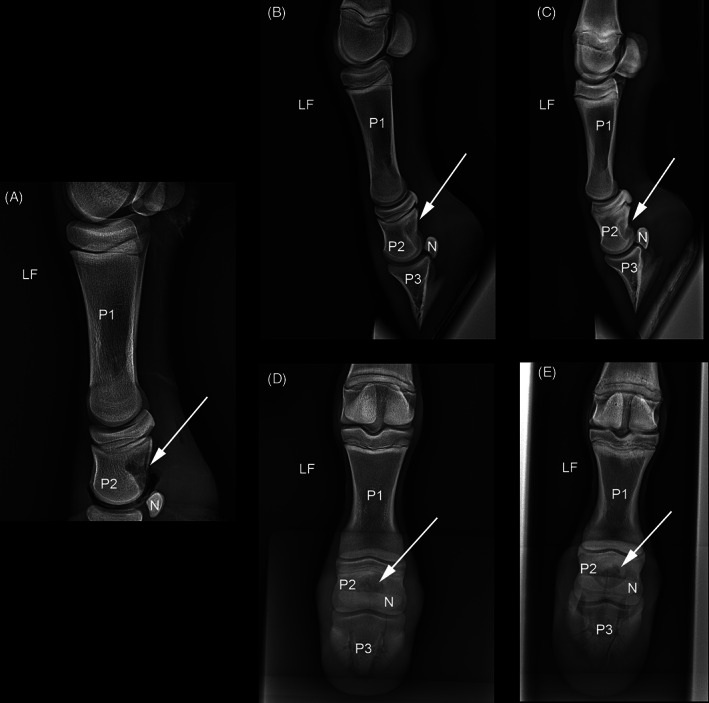
Radiographic images (68 kV, 1.8 mA) of the flexed LF distal limb intraoperatively (A), at 6 weeks (B,D), and at 20 weeks (C,E) post‐surgery illustrating the radiolucent defect after the debridement of solitary osteochondromas (arrows). LF, left thoracic limb; N, distal sesamoid bone; P1, first phalanx; P2, second phalanx; P3, third phalanx

**FIGURE 6:**
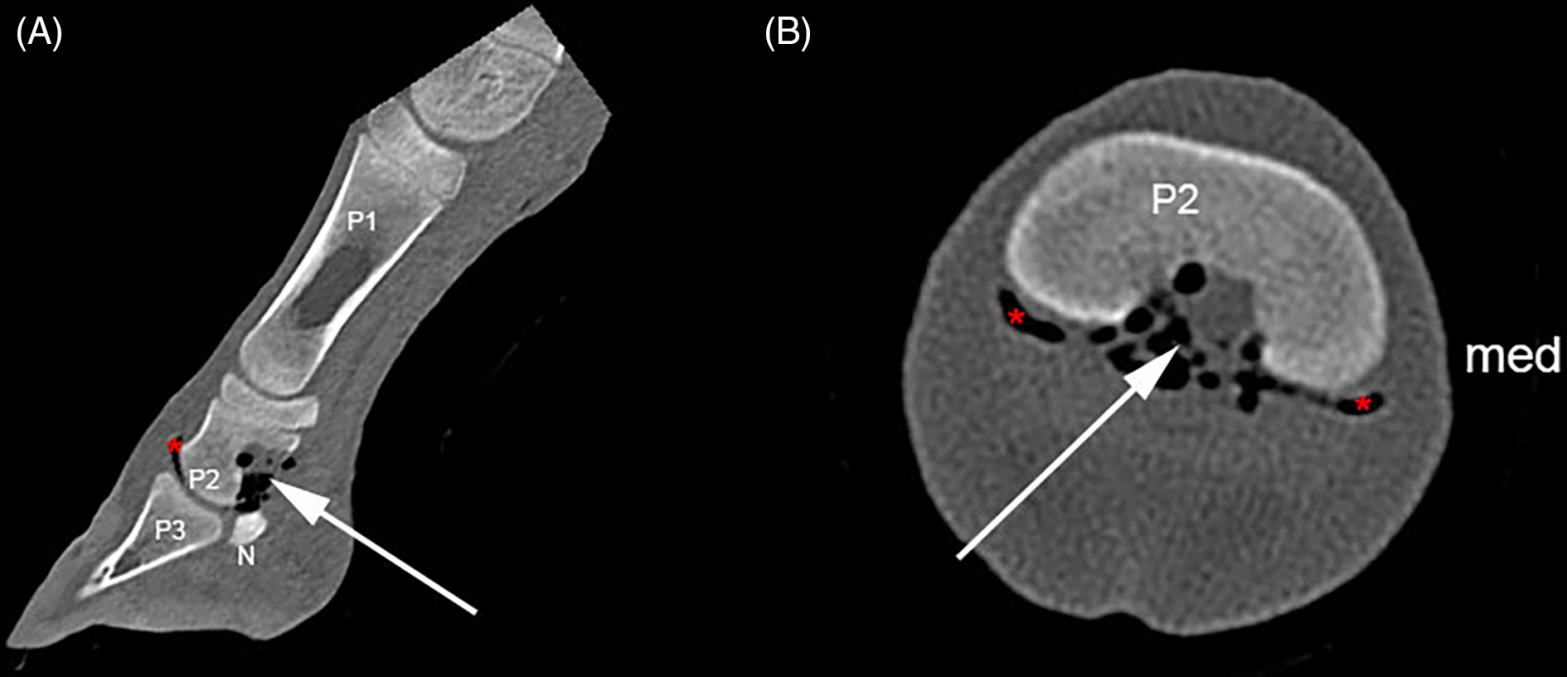
Immediate postoperative sagittal (A) and transverse (B) plane computed tomographic images. Osseous defect in P2 after debridement of osseous mass (arrows) surrounded by synovial fluid, blood clots and air. *, distal interphalangeal joint; N, distal sesamoid bone; P1, first phalanx; P2, second phalanx, P3, third phalanx

The junction between normal and abnormal bone could be easily identified at the medial and distal aspects but was difficult to distinguish laterally and proximally. Debridement was continued until healthy‐appearing margins were encountered, characterized by bleeding when irrigation was discontinued. Intraoperative radiographs were taken to confirm removal of the entire mass (Figure 5A). High‐volume arthroscopic lavage of the joint was performed, and the skin portals were closed routinely in simple interrupted (USP 2‐0 polypropylene) fashion. Intra‐articular bupivacaine (15 mg) and morphine (5 mg) were administered, and a sterile distal limb bandage was applied. Immediately postoperative CT was performed and confirmed complete removal of the osseous mass (Figure 6). Hand‐assisted recovery from anesthesia was uneventful.

### 2.8 Histopathology

**FIGURE 7:**
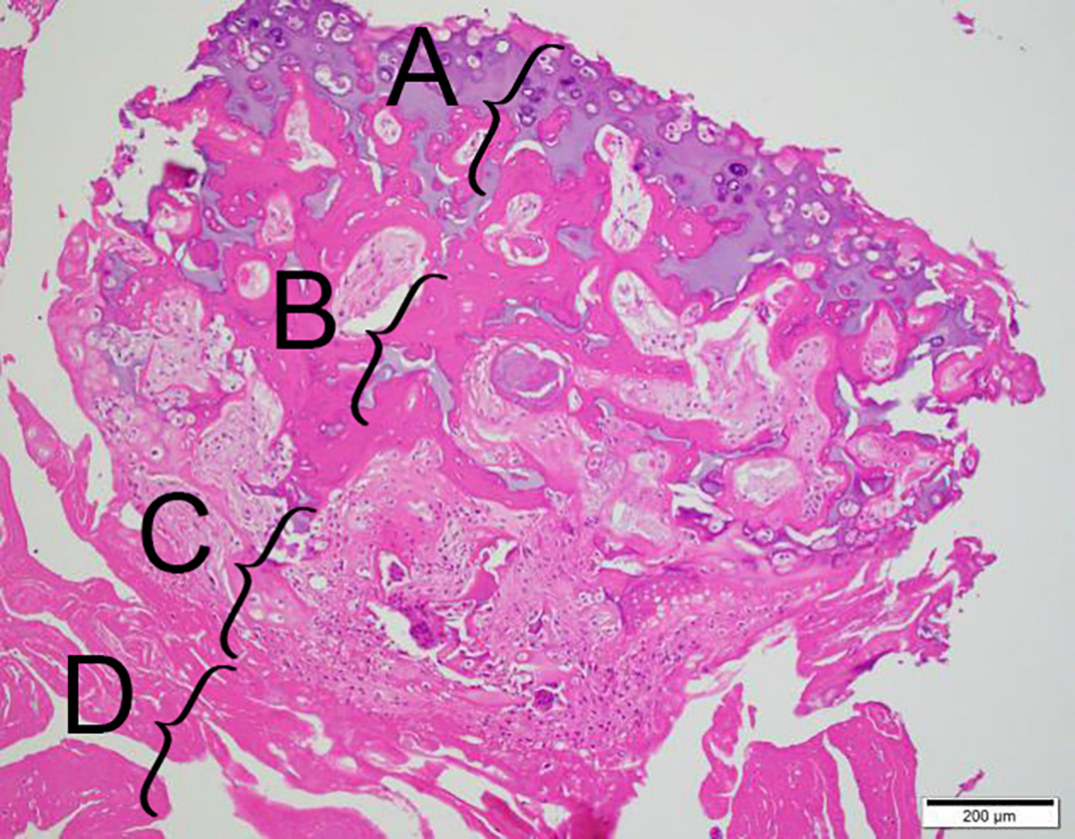
Histopathologic image of part of the osseous mass after hematoxylin and eosin staining. The upper cartilage tissue with chondrocyte clusters rich in the matrix (A), the underlying endochondral ossification forming trabecular bone (B), the lower fibrovascular tissue with foci of mineralization (C), and the lowest avascular dense subsynovial connective tissue of joint (D). Scale bar = 200 μm

Excised osseous exostoses were fixed in 10% formalin and subsequently decalcified, embedded in paraffin, and stained with hematoxylin and eosin. Microscopic examination revealed hyaline cartilage, clusters of chondrocytes, fibrovascular tissue with foci of mineralization, and signs of endochondral ossification. The tissue was continuous, with dense and avascular connective tissue. The hyperplastic chondrocytes did not express any atypical shape or mitosis, providing evidence of a benign origin. The histological architectural pattern supported a diagnosis of SO (Figure 7).^4^

## 3 RESULTS

Postoperatively, the foal received pentosan 3 mg/kg IM once, ceftiofur 2.2 mg/kg IV twice daily for 5 days, and flunixin 1.1 mg/kg IV twice daily for the first 2 days. A 12‐hour dosing interval for flunixin (24‐hour dosing interval is recommended in foals) was warranted to ensure proper analgesia in the immediate postoperative phase. Then, meloxicam 0.6 mg/kg orally once daily was administered for 1 day, followed by half doses for 2 days. Daily bandage changes were performed until day 4 postoperatively at which time the arthroscopic portals had sealed, and subsequently the bandage was changed every other day. The foal returned to full weight bearing on the affected limb 24 to 36 hours after surgery. Flexor tendon laxity resulting in fetlock drop developed progressively, likely due to bandaging. For support, a palmar heel extension was applied to the foot, and the bandage was reduced to cover only the pastern and foot region.

The colt was discharged on day 8 postsurgery, with recommendations to change the bandage every other day until day 12 (when sutures were removed) and to continue IM pentosan injections. The heel extension was left in place for 2 weeks after discharge. Box rest with 5 minutes hand walking twice daily was recommended until recheck.

The foal was rechecked at 6, 12, and 20 weeks postsurgery. By 6 weeks, the LF fetlock angle had normalized. No effusion of the DIPJ was palpable. The skin over the surgical sites was thickened, but there was normal range of motion of the phalangeal joints and absence of pain when the palmar pastern region was palpated. Mild pain was elicited by full passive digital flexion at 6 weeks, but this resolved by 12 weeks. No lameness was noted at walk or trot in a straight line on a hard surface from 6 weeks postsurgery. Small paddock turnout for short periods daily was recommended from 6 weeks postsurgery, and unrestricted exercise was allowed by 12 weeks.

**FIGURE 8:**
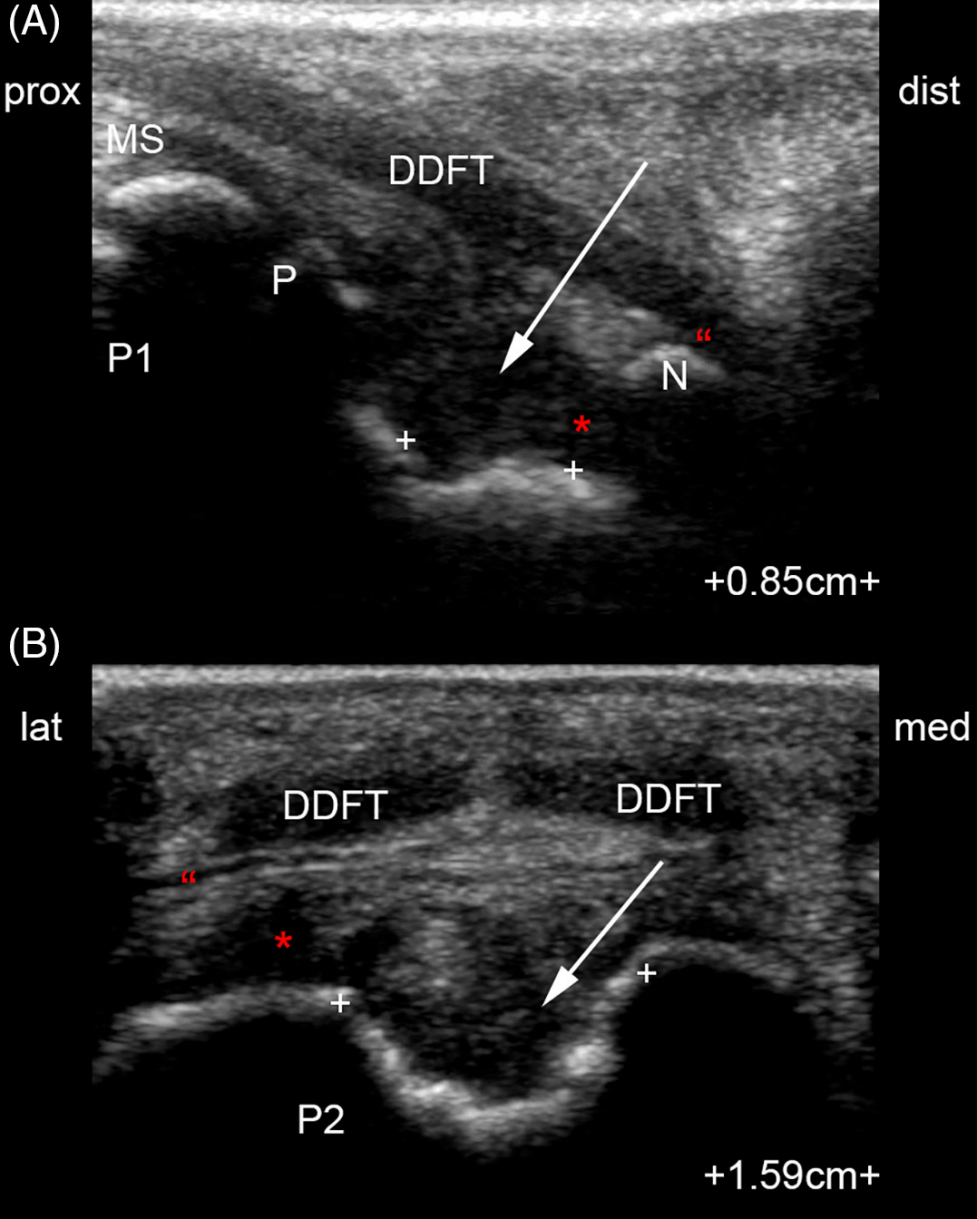
Palmar longitudinal (A) and transverse (B) ultrasound views acquired 6 weeks postoperatively. *, distal interphalangeal joint proximal palmar recess; DDFT, deep digital flexor tendon; dist, distal; lat, lateral; med, medial; MS, middle scutum; N, distal sesamoid bone; P, P2 physis; P1, first phalanx; P2, second phalanx; prox, proximal; ", navicular bursa proximal recess

Radiographs of the left thoracic limb (Figure 5B‐E) revealed that the bone defect created at the palmar aspect of P2 was progressively replaced by mineralized tissue. A rim of sclerotic bone was noted by 6 weeks postsurgery. No evidence of DIPJ osteoarthritis was seen at any point. Resolution of DIPJ and NB effusion was confirmed with ultrasound at 6 weeks postsurgery (Figure 8). The bone defect on P2 was still apparent, but had smooth margins.

## 4 DISCUSSION

Surgical debridement of an SO in a juvenile Arabian colt at the level of the mid‐diaphysis of P2 resulted in an excellent outcome. The exact nature of the osseous mass was unknown prior to surgery, but radiography and CT findings provided evidence of an SO, despite the atypical presentation.^12^

Initial differential diagnoses included congenital malformation, exostosis, and malignant osteochondral tumors. The location can be used to differentiate between true exostosis and SO.^4^ A true exostosis is located at the physeal scar, while SO drift progressively toward the diaphysis following longitudinal bone growth. A defining feature of SO is its continuity with the underlying cortex and, in some cases, with the medullary cavity.^3^ On the basis of the pathogenesis, Murphey et al^10^ suggested that SO should be classified as developmental lesions, not benign tumors. Unlike in man and in dogs, malignant transformations into chondrosarcoma or osteosarcoma have not been reported in horses.^3^, ^13^ In our case, the connection between the SO and cortex and medulla of P2 was confirmed by CT. Malignancy of the mass was unknown prior to surgery; this highlights the importance of a complete surgical debridement and histopathologic examination.

Bilateral radial SO^13^ and multiple hereditary exostosis (HME) have been described in horses. Multiple hereditary exostosis is a rare autosomal dominant genetic disorder characterized by the development of multiple benign osteocartilaginous masses on the skeleton.^14^, ^15^ Therefore, the areas where bone growth is most active and subject to stress (including ribs) were scrutinized in our colt and found to be free from palpable osseous protrusions. There was no evidence of bilateral SO or HME in this case, which simplified treatment and improved the colt's reproductive prognosis.

On the basis of the initial evaluation, SO was considered the most likely diagnosis. Computed tomography allowed thorough presurgical planning, which included mapping the mass in detail, anticipating the potential for soft tissue damage, and selecting a minimally invasive arthroscopic approach over an open approach. Because of risk of postoperative sepsis and surgical trauma to the surrounding structures, an open approach was not considered in this foal. The lateral/medial approach, similar to that described by Fowlie et al,^11^ was elected over the original palmar DIPJ arthroscopic technique described by Vacek et al.^16^ The portals were located abaxial to the neurovascular digital bundles, consequently reducing the risk of inadvertent penetration of the distended DFTS and NB by 50%. The use of a 2.7‐mm OD arthroscope may have improved maneuverability within the joint; however, a technical failure forced us to use a 4‐mm OD arthroscope. The margins of the osseous mass became difficult to ascertain intraoperatively while the motorized bone burr was used because of very little tactile feedback during the mechanical debridement. This created a higher risk of excessively removing healthy tissue. An alternative was manual debridement; however, this would have been time consuming and would have required a larger instrument portal. The generation of large quantities of osteochondral debris by the motorized bone burr was addressed by use of a higher irrigation rate than usual as well as frequent high‐volume lavages to flush the remaining debris. Regular close observation of the debridement bed with arthroscopic magnification was performed when the irrigation was reduced. The mass made of compact white bone was relatively deprived of small blood vessels, but these became apparent after the normal cancellous, yellow/pink bone had been reached. A complete debridement was considered imperative because it was unknown at the time whether the mass was malignant or benign.

This SO originating from P2 growth plate impinged markedly on the suspensory apparatus of DSB and caused severe lameness in this foal. It was associated with a marked nonseptic inflammation of the DIPJ and should be considered as a differential in juveniles. Computed tomography‐arthrography played a key role in presurgical planning and histopathology for diagnostic confirmation. In contrast to what was described by Easter et al^7^ in 1998, arthroscopic debridement through a modified lateral/medial palmar approach of the palmar proximal recess of the DIPJ with motorized equipment allowed extirpation of all the abnormal bone. An excellent outcome was obtained with complete resolution of lameness and DIPJ inflammation by 6 weeks postoperatively. Although long‐term follow up is required, the weakened palmar cortex of P2 will likely fully heal by the time the horse enters athletic activity.

## AUTHOR CONTRIBUTIONS

Ysebaert MP, DVM: Acquired and analyzed clinical data, prepared illustrations, drafted the manuscript, and critically revised the manuscript final version; Johnson JP, DVM, DECVS, DVMS: Acquired and analyzed clinical data and critically revised the manuscript final version; Abbas G, DVM: Acquired clinical data and critically revised the manuscript final version; Cavalcante PH, DVM: Acquired clinical data and critically revised the manuscript final version; King R, AWCF: Acquired clinical data and critically revised the manuscript final version; Oikawa M, DVM, DJCVP, PhD: Acquired clinical data and critically revised the manuscript final version; Puchalski S, DVM, DACVR: Acquired and analyzed clinical data and critically revised the manuscript final version; David F, DVM, DACVS, DECVS, DACVSMR, DECVSMR, ECVDI Assoc.: Designed original work, acquired and analyzed clinical data, prepared illustrations, and critically revised the final manuscript version.

## CONFLICT OF INTEREST

The authors declare no conflicts of interest related to this report.

## SUPPORTING INFORMATION

Additional supporting information may be found online in the Supporting Information section at the end of this article.

## Supporting information

**AppendixS1:** Supporting information
